# Complete Genome Sequence of *Chlamydia trachomatis* Ocular Serovar C Strain TW-3

**DOI:** 10.1128/genomeA.01204-13

**Published:** 2014-01-23

**Authors:** Vítor Borges, Miguel Pinheiro, Luís Vieira, Daniel A. Sampaio, Alexandra Nunes, Maria J. Borrego, João P. Gomes

**Affiliations:** a Reference Laboratory of Bacterial Sexually Transmitted Infections, Department of Infectious Diseases, National Institute of Health, Lisbon, Portugal; b Bioinformatics Unit, Department of Infectious Diseases, National Institute of Health, Lisbon, Portugal; c School of Medicine, University of St. Andrews, Fife, United Kingdom; d Innovation and Technology Unit, Human Genetics Department, National Institute of Health, Lisbon, Portugal

## Abstract

*Chlamydia trachomatis* is the etiological agent of trachoma, the leading infectious cause of blindness worldwide. We report here the first complete and annotated genome of a *C. trachomatis* trachoma-causing serovar C strain (strain TW-3). The chromosome and plasmid are 1,043,554 bp and 7,501 bp in length, respectively.

## GENOME ANNOUNCEMENT

The obligate intracellular bacterium *Chlamydia trachomatis* is the major cause of bacterial sexually transmitted infections worldwide and is also responsible for trachoma, the leading infectious cause of blindness. Trachoma results from a conjunctival chronic inflammatory state leading to the formation of irreversible corneal opacities and blindness (1, 2). It is one of 17 neglected tropical diseases and is targeted for elimination by 2020 by the World Health Organization (WHO) through the implementation of the SAFE strategy: lid surgery (S), antibiotics to treat the infection (A), facial cleanliness (F), and environmental changes (E) (3). Trachoma affects about 2.2 million people and it is endemic in >50 countries, predominantly in sub-Saharan Africa, the Middle East, and Asia (2). This epidemiological pattern is essentially associated with *C. trachomatis* ocular serovars A and B (4) (from the existent 15 major serovars), whereas serovar C seems relatively common in indigenous Australian communities (5, 6). Of note, serovar C has been also associated with *Chlamydia*-related arthritis (7). There are already five and two annotated genomes from serovars A and B (8–10), respectively. We report here the first complete and annotated sequence of a trachoma-causing *C. trachomatis* serovar C strain, TW-3.

The C/TW-3 strain was isolated in Taiwan in 1959 from the human conjunctiva (11). We obtained this strain from the American Type Culture Collection (ATCC VR-1477) and propagated it in HeLa229 cell monolayers before proceeding with bacterial purification using discontinuous urographin density gradients (12). The whole-genome sequence was determined by using a paired-end strategy (2 × 250 bp) with the platform Illumina MiSeq. The reads were mapped to *C. trachomatis* chromosome and plasmid sequences (8–10) using both Bowtie 2 (version 2.1.0 [http://bowtie-bio.sourceforge.net/bowtie2/index.shtml]) (13) and the Burrows-Wheeler Aligner (BWA) software (version 0.7.5a [http://bio-bwa.sourceforge.net/]) (14). Globally, 5,233,958 reads (with a mean quality score >30 for >95% of the read bases) were mapped, which yielded a mean coverage of 1,117-fold and 6,717-fold for the chromosome and plasmid, respectively. Single-nucleotide polymorphisms (SNPs) and indels were identified using SAMtools, followed by variant calling using BCFtools (http://samtools.sourceforge.net/) (15), and were carefully inspected through the Integrative Genomics Viewer (version 2.3.12 [http://www.broadinstitute.org/igv/]) (16). Both the typing gene (*ompA*) and problematic regions (e.g., *tarP*) were confirmed by PCR, followed by Sanger sequencing. The sequence was annotated by the NCBI Prokaryotic Genomes Annotation Pipeline 2.3.

The genome sequence of C/TW-3 revealed a chromosome of 1,043,554 bp in length, with a G+C content of 41.30% and 922 predicted coding sequences (CDSs). Plasmid analysis revealed the existence of about six copies per chromosome (based on the ratio of mean coverage for plasmid/chromosome), which fits with previous data (17), and the plasmid was found to be 7,501 bp in length, comprising eight CDSs (G+C content of 36.25%).

The availability of a complete and annotated genome sequence of an additional trachoma-causing serovar may contribute to the elucidation of the genetic basis underlying the pathogenic differences between *C. trachomatis* ocular strains (7, 18, 19).

### Nucleotide sequence accession numbers.

The complete genome sequence of *C. trachomatis* serovar C (strain TW-3) has been assigned GenBank accession no. CP006945 (chromosome) and CP006946 (plasmid).

## References

[1] Burton MJ Mabey DC 2009 The global burden of trachoma: a review. PLOS Negl. Trop. Dis. 3:e460. 10.1371/journal.pntd.0000460 19859534 PMC2761540

[2] World Health Organization 2012 Global WHO alliance for the elimination of blinding trachoma by 2020. Wkly. Epidemiol. Rec. 87:161–168 WHO, Geneva, Switzerland http://www.who.int/wer/2012/wer8717.pdf 22574352

[3] World Health Organization 1998 Global elimination of blinding trachoma. Resolution WHA 51.11 adopted by World Health Assembly. 16 May 1998, World Health Organization, Geneva, Switzerland

[4] Andreasen AA Burton MJ Holland MJ Polley S Faal N Mabey DC Bailey RL 2008 *Chlamydia trachomatis ompA* variants in trachoma: what do they tell us? PLOS Negl. Trop. Dis. 2:e306. 10.1371/journal.pntd.0000306 18820750 PMC2553491

[5] Stevens MP Tabrizi SN Muller R Krause V Garland SM 2004 Characterization of *Chlamydia trachomatis* omp1 genotypes detected in eye swab samples from remote Australian communities. J. Clin. Microbiol. 42:2501–2507. 10.1128/JCM.42.6.2501-2507.2004 15184427 PMC427869

[6] Porter M Mak D Chidlow G Harnett GB Smith DW 2008 The molecular epidemiology of ocular *Chlamydia trachomatis* infections in western Australia: implications for trachoma control. Am. J. Trop. Med. Hyg. 78:514–517 18337352

[7] Gerard HC Stanich JA Whittum-Hudson JA Schumacher HR Carter JD Hudson AP 2010 Patients with *Chlamydia*-associated arthritis have ocular (trachoma), not genital, serovars of *C. trachomatis* in synovial tissue. Microb. Pathog. 48:62–68. 10.1016/j.micpath.2009.11.004 19931374 PMC2815210

[8] Carlson JH Porcella SF McClarty G Caldwell HD 2005 Comparative genomic analysis of *Chlamydia trachomatis* oculotropic and genitotropic strains. Infect. Immun. 73:6407–6418. 10.1128/IAI.73.10.6407-6418.2005 16177312 PMC1230933

[9] Seth-Smith HM Harris SR Persson K Marsh P Barron A Bignell A Bjartling C Clark L Cutcliffe LT Lambden PR Lennard N Lockey SJ Quail MA Salim O Skilton RJ Wang Y Holland MJ Parkhill J Thomson NR Clarke IN 2009 Co-evolution of genomes and plasmids within *Chlamydia trachomatis* and the emergence in Sweden of a new variant strain. BMC Genomics 10:239. 10.1186/1471-2164-10-239 19460133 PMC2693142

[10] Harris SR Clarke IN Seth-Smith HM Solomon AW Cutcliffe LT Marsh P Skilton RJ Holland MJ Mabey D Peeling RW Lewis DA Spratt BG Unemo M Persson K Bjartling C Brunham R de Vries HJ Morré SA Speksnijder A Bébéar CM Clerc M de Barbeyrac B Parkhill J Thomson NR 2012 Whole-genome analysis of diverse *Chlamydia trachomatis* strains identifies phylogenetic relationships masked by current clinical typing. Nat. Genet. 44:413–419, S1. 10.1038/ng.2214 22406642 PMC3378690

[11] Grayston JT Wang SP Woolridge RL Yang YF Johnston PB 1960 Trachoma-studies of etiology, laboratory diagnosis, and prevention. JAMA 172:1577–1586. 10.1001/jama.1960.03020150001001 13829148

[12] Borges V Ferreira R Nunes A Sousa-Uva M Abreu M Borrego MJ Gomes JP 2013 Effect of long-term laboratory propagation on *Chlamydia trachomatis* genome dynamics. Infect. Genet. Evol. 17:23–32. 10.1016/j.meegid.2013.03.035 23542454

[13] Langmead B Salzberg SL 2012 Fast gapped-read alignment with Bowtie 2. Nat. Methods 9:357–359. 10.1038/nmeth.1923 22388286 PMC3322381

[14] Li H Durbin R 2010 Fast and accurate long-read alignment with Burrows-Wheeler transform. Bioinformatics 26:589–595. 10.1093/bioinformatics/btp698 20080505 PMC2828108

[15] Li H Handsaker B Wysoker A Fennell T Ruan J Homer N Marth G Abecasis G Durbin R 1000 Genome Project Data Processing Subgroup 2009 The Sequence Alignment/map format and SAMtools. Bioinformatics 25:2078–2079. 10.1093/bioinformatics/btp352 19505943 PMC2723002

[16] Thorvaldsdóttir H Robinson JT Mesirov JP 2013 Integrative Genomics viewer (IGV): high-performance genomics data visualization and exploration. Brief. Bioinform. 14:178–192. 10.1093/bib/bbs017 22517427 PMC3603213

[17] Ferreira R Borges V Nunes A Borrego MJ Gomes JP 2013 Assessment of the load and transcriptional dynamics of *Chlamydia trachomatis* plasmid according to strains’ tissue tropism. Microbiol. Res. 168:333–339. 10.1016/j.micres.2013.02.001 23590987

[18] Borges V Nunes A Ferreira R Borrego MJ Gomes JP 2012 Directional evolution of *Chlamydia trachomatis* towards niche-specific adaptation. J. Bacteriol. 194:6143–6153. 10.1128/JB.01291-12 22961851 PMC3486361

[19] Kari L Whitmire WM Carlson JH Crane DD Reveneau N Nelson DE Mabey DC Bailey RL Holland MJ McClarty G Caldwell HD 2008 Pathogenic diversity among *Chlamydia trachomatis* ocular strains in nonhuman primates is affected by subtle genomic variations. J. Infect. Dis. 197:449–456. 10.1086/525285 18199030

